# Corollary discharge dysfunction in auditory verbal hallucinations: N100 suppression measured with semi-dry electrodes

**DOI:** 10.1016/j.cnp.2026.10.001

**Published:** 2026-10-06

**Authors:** Ella Sonnelid, Robert Boden, Caroline Wass, Jonas Persson

**Affiliations:** a Department of Medical Sciences, Clinical Psychiatry, Uppsala University, Akademiska sjukhuset, ingång 10, plan 3, 751 85 Uppsala, Sweden; b Department of Psychiatry for Affective Disorders, Sahlgrenska University Hospital, Region Västra Götaland, Sahlgrenska Universitetssjukhuset Psykiatrimott Gamlestaden, 413 45 Göteborg, Sweden

**Keywords:** Corollary discharge, Schizophrenia, Event related potential, Semi-dry electrodes, Electroencephalography, Attenuation

## Abstract

**Objective:**

Corollary discharge dysfunction in patients with auditory verbal hallucinations (AVH) is reflected in failure to attenuate the N100 event-related potential. Previous research has shown mixed findings regarding N100 suppression and hallucination severity, generally using broad symptom scales rather than AVH-specific measures. This study investigated corollary discharge and its relationship with comprehensively characterized auditory hallucinations.

**Methods:**

Corollary discharge was investigated in 14 patients with persistent auditory hallucinations and 15 healthy controls using an EEG paradigm using semi-dry electrodes where participants self-generated sounds in a “talk” condition and listened to playback in a “listen” condition. N100 suppression was calculated as the peak-amplitude difference (μV) between talk and listen in the 60–150 ms window. Auditory hallucinations were assessed with the Psychotic Symptom Rating Scales Auditory Hallucination Subscale (PSYRATS-AHS).

**Results:**

Between-group analysis of the fronto-central ROI (Fz-Cz) showed a statistically non-significant trend toward greater N100 suppression in healthy controls compared to AVH patients (*p* = 0.095, Cohen's d = 0.64). N100 suppression showed significant negative correlation with hallucination severity in the AVH group (*r* = −0.54, *p* = 0.047).

**Conclusions:**

Hallucination severity is related to impaired corollary discharge mechanisms, supporting its role as a mechanistic basis for auditory hallucinations.

**Significance:**

The preliminary correlation with PSYRATS-AHS suggests potential value in using symptom-specific assessment tools in research on auditory hallucinations, warranting replication in larger samples.

## Highlights

• N100 suppression correlates with auditory verbal hallucination (AVH) severity.
• AVH-specific rating scales may better capture N100-symptom relationships.
• Reduced N100 suppression in AVH patients (d = 0.64) is consistent with prior literature.

## 1 Introduction

Auditory verbal hallucinations (AVH) are commonly experienced among people with schizophrenia and schizophrenia spectrum disorders. Around 60–80% of people diagnosed with these disorders are affected by AVH (Lim et al., 2016). This symptom can lead to great suffering and functional impairment in the daily lives of affected individuals.

Corollary discharge refers to a copy of motor commands that is sent to sensory areas of the brain during voluntary movements, preparing these areas for the sensory consequences of the planned action. In speech production, this efference copy involves the motor cortex sending a signal to the auditory cortex to prepare it for self-generated auditory stimuli. When the auditory cortex receives this preparatory signal, self-generated sounds are categorized as such and their perception is suppressed (Ford and Mathalon, 2005; Thoma et al., 2017). However, when this mechanism malfunctions or is absent, individuals may perceive self-generated auditory stimuli similarly to external sounds, creating difficulty in distinguishing between internal and external auditory experiences (Parlikar et al., 2019; Poletti et al., 2019). This failure to produce adequate corollary discharge has been proposed as a mechanistic basis of auditory verbal hallucinations (Parlikar et al., 2019; van Lutterveld et al., 2011).

To study the neurological mechanism of corollary discharge, researchers commonly use electroencephalography (EEG) and event-related potential (ERP) analysis, which offers excellent temporal resolution and allows for capturing this mechanism in real time. Within the EEG recording, a component expressed as a negativity around 100 ms post-presented auditory stimuli has been linked to this mechanism (Bühler et al., 2016; Ford et al., 2010; Nawani et al., 2014). A failure to attenuate this component reflects disrupted corollary discharge (Ford et al., 2007a; Ford et al., 2010 Ford and Mathalon, 2008; Samantaray et al., 2025). When the corollary discharge mechanism exhibits normal functionality, the response in the brain should be attenuated and the N100 component should be closer to zero microvolts. Therefore, N100 suppression, calculated as the amplitude difference between self-generated and externally presented sounds, serves as a validated measure of corollary discharge function (Beño-Ruiz-de-la-Sierra et al., 2023a; Ford, 2016; Ford et al., 2021; Roach et al., 2020).

Regions of interest in corollary discharge research are typically the fronto-central regions (Beño-Ruiz-de-la-Sierra et al., 2023; Ford et al., 2016). The underlying brain regions involved in these mechanisms are believed to be the fronto-striatal circuits, as well as the pre-motor areas implicated in corollary discharge and auditory cortex signaling processes relevant to auditory verbal hallucinations (Heinks-Maldonado et al., 2007; Ford et al., 2002; Schneider et al., 2014). Based on their anatomical placement over the supplementary motor area (SMA) and the recent focus on fronto-central electrodes in corollary discharge research (Ford et al., 2021), Fz and Cz electrodes were selected for the region of interest in the N100 analysis.

The use of EEG devices is generally accessible, both regarding cost and effort. However, the wet-electrodes often used in research settings present challenges such as tedious setup to gain adequate connection between the electrodes and scalp and to achieve low impedance, often around <5kΩ (Kappenman and Luck, 2010). This process can become a hurdle for researchers, and when not implemented properly, results in suboptimal data quality. Alternatives to wet electrodes are semi-dry and dry electrodes. Semi-dry electrodes typically use saline solutions on cotton mediums to establish connection between the electrodes and the scalp. This setup allows for a less demanding and quicker setup, increasing the accessibility of this modality for researchers working with patient populations that benefit from shorter preparation times, as well as researchers with less experience working with EEG (Li et al., 2020).

To capture the symptom of AVH, general rating scales provide a broad picture of the symptom profile in patients with schizophrenia or schizophrenic spectrum disorders. However, there may be benefit to focusing on the symptom itself rather than the disorder, since different etiologies may present with very homogenous symptoms, in this case AVH. Therefore, finding a more targeted rating scale is key to effectively understanding AVH, regardless of the diagnosis under which it presents, and to finding treatments that target this specific symptom. The Psychotic Symptom Rating Scales (PSYRATS), and especially the Auditory Hallucination Subscale (PSYRATS-AHS), is a promising tool to capture the symptoms of AVH more directly than the alternative rating scales used in schizophrenia and schizophrenia spectrum disorders (Ford, 2016). This scale captures multiple dimensions of the symptom, such as duration and nature of hallucinations experienced, that may be crucial to gaining more complete insight into AVH.

Previous studies have consistently demonstrated reduced N100 suppression in patients with schizophrenia compared to healthy controls (Baess et al., 2011; Beño-Ruiz-de-la-Sierra et al., 2023b; Ford et al., 2001; Ford et al., 2007b; Ford and Mathalon, 2004). However, findings regarding the relationship between N100 suppression and AVH severity have been mixed, with some studies reporting significant correlations (Ford et al., 2007) while others found no such relationship (Ford et al., 2007a, Ford & Mathalon, 2007). The use of broader rating scales might explain why previous studies have found inconsistent results when attempting to correlate rating scale scores with N100 suppression. AVH-specific rating scales such as the PSYRATS-AHS may be more sensitive to detecting relationships with neurophysiological markers by capturing detailed phenomenological dimensions of auditory hallucinations (Ford, 2016). Additionally, the more widespread adoption of AVH-specific rating scales could help ensure comparable findings across studies.

The main aim of this study was to investigate the corollary discharge mechanism and its relationship with comprehensively characterized auditory hallucinations using the PSYRATS-AHS.

## 2 Materials and methods

### 2.1 Trial design and ethical standards

Data for this study were derived from a clinical trial registered at clinicaltrials.gov (ID: NCT04651621). The EEG recordings included in this study were collected during a vocalization task conducted at the baseline timepoint prior to therapeutic intervention. The study was approved by the Swedish ethical review board (DNR 2019–06467) and conducted in compliance with the Declaration of Helsinki (1975, revised 2008).

### 2.2 Participants

Twenty patients were recruited from the psychiatric outpatient clinics at Uppsala University Hospital in Sweden and the study was conducted at the hospital. Inclusion criteria were 18–59 years of age, daily AVH occurrence for at least one month prior to study inclusion, stable pharmacotherapy one month prior to inclusion, and written informed consent. Exclusion criteria included contraindications to rTMS treatment, ongoing substance abuse disorder assessed by the Drug Use Disorders Identification Test (DUDIT) and urine screen, alcohol use disorder assessed by the Alcohol Use Disorders Identification Test (AUDIT) and BPEth, pregnancy, or other circumstances likely to hinder participation in the study.

Out of the 20 patients with auditory verbal hallucinations (AVH) originally recruited for the study, three participants were lost due to technical issues in data acquisition, and additionally three were lost due to excessive artifact occurrence. Out of the 20 healthy control participants (HC) originally recruited five were lost due to technical issues in data acquisition. Technical problems leading to exclusion at the acquisition stage were primarily related to unreliable trigger box connections and cabling, which resulted in missing triggers required for talk/listen epoching, while artifact-related exclusions reflected data quality issues identified during preprocessing (see section 2.4.2 for details). An overview of the study design and participant flow is presented in Supplementary Fig. S1.

### 2.3 Rating scales

Patients' auditory verbal hallucination symptoms were assessed using the PSYRATS. PSYRATS is comprised of 17 items split into two subscales, the Auditory Hallucination Subscale (AHS) and the Delusion Subscale (DS). In the present study, the Auditory Hallucination Subscale consisting of the initial 11 questions of the questionnaire was of interest, referred to as PSYRATS-AHS. Each of the 11 items is rated on a scale from 0 (absent) to 4 (severe). The PSYRATS-AHS captures detailed phenomenological characteristics of auditory hallucinations, including frequency, loudness, and spatial location, providing comprehensive assessment beyond what is available in other rating scales (Woodward et al., 2014). The PSYRATS has demonstrated excellent inter-rater reliability and good internal consistency in patients with schizophrenia spectrum disorders (Kronmüller et al., 2011). Alcohol Use Disorder Identification Test (AUDIT) and Drug Use Disorder Identification Test (DUDIT) were used to screen for substance use disorder. EuroQol Visual Analogue Scale (EQ-VAS) is a self-reported of global health from 0 to 100. Clinical interviews were done the day of the ERP testing by a trained clinician (RB).

### 2.4 Data acquisition and preprocessing

Data consisted of a 32-channel EEG recorded using an Easycap EEG-cap with semi-dry active electrodes positioned in a 10–20 system (Supplementary Fig. S2) and a TMSi SAGA 32+/64+ amplifier system (TMSi B.V., Oldenzaal, Netherlands). The online reference was the average of all electrodes, and the ground was placed on the nose. The channels included Fp1, Fpz, Fp2, F7, F3, Fz, F4, F8, FC5, FC1, FC2, FC6, T7, C3, Cz, C4, T8, CP5, CP1, CP2, CP6, P7, P3, Pz, P4, P8, POz, O1, Oz, O2, M1, M2. The impedances were kept below 50 kΩ and data recorded at a sampling rate of 2000 Hz.

#### 2.4.1 The say-ah paradigm

The *Say-Ah* paradigm was set up in accordance with prior literature (Ford et al., 2010). The participant was placed in a comfortable chair in front of a screen. Instructions were to watch the screen and generate an “ah” sound each time a marker appeared on the screen. Firstly, a training session was performed ensuring that the participant produced the “ah” sound with minimal facial movements in order to minimize EEG artifacts, although loud enough to register as a marker in the recording. Thereafter, a total of 20 “ah” sounds were recorded in the “talk” condition, and subsequently played back to the participant through headphones in the “listen” condition. This sequence of “talk” followed by “listen” were repeated over five sessions, resulting in 100 events in the “talk” condition and 100 events in the “listen” condition. EEG was continuously recorded during the paradigm, and a trigger code was entered into the EEG at the onset of each speech sound (“ah”), spoken and heard, to enable epoching of the EEG signal, phase locked to onset of the event. The total paradigm duration was around 5 min.

#### 2.4.2 Data processing

Preprocessing was performed using EEGLAB v2024.2.1 and Matlab R2024b (MathWorks Inc., MA, USA). Continuous data were downsampled to 512 Hz, FIR high-pass filtered at 0.5 Hz, and notch-filtered (48–52 Hz) to remove line noise. Artifact Subspace Reconstruction (ASR) was applied using default parameters from an optimized pipeline for EEGLAB (Delorme, 2023; Nolan et al., 2010). Independent Component Analysis (ICA) (Delorme et al., 2007) and ICLabel were used to identify and reject artifacts, with a threshold of 0.8 for both “eye” and “muscle” components. Offline re-referencing used the average of all electrodes due to excessive artifacts at the mastoids (M1, M2) caused by the vocalization task. Data were then epoched from −100 ms to 400 ms around events and band-pass filtered at 0.5 to 15 Hz for analysis. Threshold-based rejection was applied to epochs with amplitudes exceeding ±80 μV to remove major artifacts. Channel-specific visual inspection was performed for the channels included in the region of interest, Fz and Cz, to prevent epoch rejection based on artifacts in channels outside the region. Any remaining trials with artifacts in these target channels were manually removed. Participants with fewer than 20% of trials remaining in either condition were excluded to ensure stable ERP estimation, resulting in three excluded participants. The exclusion of data at this stage was due to offline processing revealing elevated noise levels. The “talk” condition was consistently noisier than “listen” across all timepoints and both groups; as impedance was consistent across conditions within each recording, this pattern is more likely attributable to vocalization-related muscle artifacts than to the semi-dry electrodes, resulting in a higher proportion of excluded epochs for the “talk” condition specifically. However, the higher impedances overall might have led to our recordings being more sensitive to noise and potentially picking up more of the muscle artifacts.

#### 2.4.3 N100 analysis + region of interest

N100 was defined as the amplitude at the most negative peak within the 60–150 ms window following talk or listen events. Preprocessed epochs that passed quality control were averaged per condition and electrode to create individual ERPs for Fz and Cz. Peak amplitude and latency were extracted from each electrode separately using ERPLAB. The data quality of the peak amplitudes was assessed using bootstrapped standardized measurement error (bSME) (Luck et al., 2021). A weighted ROI amplitude was computed for both conditions, talk and listen, using the formula:

$$\mathrm{RO}I_{\mathrm{peak}\_\mathrm{amp}}=\frac{Fz_{\mathrm{peak}\_\mathrm{amp}}\times Fz_{\mathrm{epochs}}+Cz_{\mathrm{peak}\_\mathrm{amp}}\times Cz_{\mathrm{epochs}}}{Fz_{\mathrm{epochs}}+Cz_{\mathrm{epochs}}}$$

Individual suppression scores were calculated as the difference between weighted talk and listen ROI amplitudes (talk - listen).

### 2.5 Statistical analysis

All statistical analyses were performed using R (version 4.5.1). Statistical significance was set at α = 0.05 for all tests.

Demographic differences between AVH and HC groups were assessed using independent samples *t*-tests for age and chi-squared tests for gender distribution.

N100 suppression scores at the weighted ROI were tested for normality using the Shapiro-Wilk test in each group. As both groups met normality assumptions, independent samples *t*-tests were used for between-group comparisons of N100 suppression. Effect size was reported using Cohen's d.

Within the AVH group, Pearson's correlation was used to examine the relationship between N100 suppression and PSYRATS-AHS total scores.

## 3 Results

The final cohort included in analysis consisted of 14 AVH patients (6 females, 8 males; mean age 32.8 ± 7.8 years) and 15 healthy controls (10 females, 5 males; mean age 28.8 ± 8.0 years) (Table 1). Groups did not differ significantly in age (*t* = 1.17, *p* = 0.254) or gender distribution (Fisher's exact test, *p* = 0.272).

**Table 1:** Sociodemographic and Clinical Characteristics of Participants.

	AVH *n* = 14	Healthy Controls *n* = 15
Sex (M/F)	8/6	5/10
Age, (years)	32.8 (7.8)	28.8 (8.0)
Handedness (R/L)	14/0	15/0
Illness duration, (years)	10.7 (7.1)	–

Worked/studied past 6 months, n (%)		
Yes	5 (35.7)	15 (100)
No	9 (64.3)	0 (0)

Education, n (%)		
University degree	2 (14.3)	5 (33.3)
High school	9 (64.3)	10 (66.7)
Primary school	2 (14.3)	0 (0)
Incomplete primary	1 (7.1)	**0 (0)**
AUDIT	2.9 (2.8)	5.5 (2.3)
DUDIT	0.0 (0.0)	0.5 (1.4)
PSYRATS	36.5 (10.2)	–
PSYRATS-AHS	29.3 (6.1)	–
Hallucination duration (years)	11.1 (7.2)	–
EQ-VAS	53.6 (18.6)	**86.1 (6.0)**

*Abbreviations*: AUDIT, Alcohol Use Disorder Identification Test; DUDIT, Drug Use Disorder Identification Test; PSYRATS, Psychotic Symptom Rating Scales; PSYRATS-AHS, Psychotic Symptom Rating Scales Auditory Hallucinations Subscale; EQ-VAS, EuroQol Visual Analogue Scale.

Epoch counts averaged 67 ± 24 for Fz and 71 ± 25 for Cz in the listen condition, and 59 ± 28 for Fz and 61 ± 26 for Cz in the talk condition. No significant differences were found between electrodes within each condition or subject groups (all *p* > 0.05).

### 3.1 Data quality

The root mean square bSME (RMS(bSME)) for N100 peak amplitude (60–150 ms window) was 0.635 μV for listen condition and 0.918 μV for talk condition at the ROI. Signal-to-measurement error ratios (N100 amplitude/bSME) averaged 3.1 for listen and 2.2 for talk conditions. The data quality proved to be better within the listen condition for both groups, as expected with the vocalization factor of the talk condition.

### 3.2 Between group comparisons

Both AVH (W = 0.95, *p* = 0.58) and HC (W = 0.96, *p* = 0.67) groups showed normal distributions, hence independent samples *t*-tests were used for between-group comparisons. Grand average ERPs are shown in Fig. 1a and b. Between group Student's *t*-tests showed that N100 suppression was greater in the HC group (M = 1.43 μV, SD = 2.49) than in the AVH group (M = 0.10 μV, SD = 1.54), though this between-group difference did not reach statistical significance (*t* = 1.74, *p* = 0.095, Cohen's d = 0.64) (Fig. 1c).

**Fig. 1:**
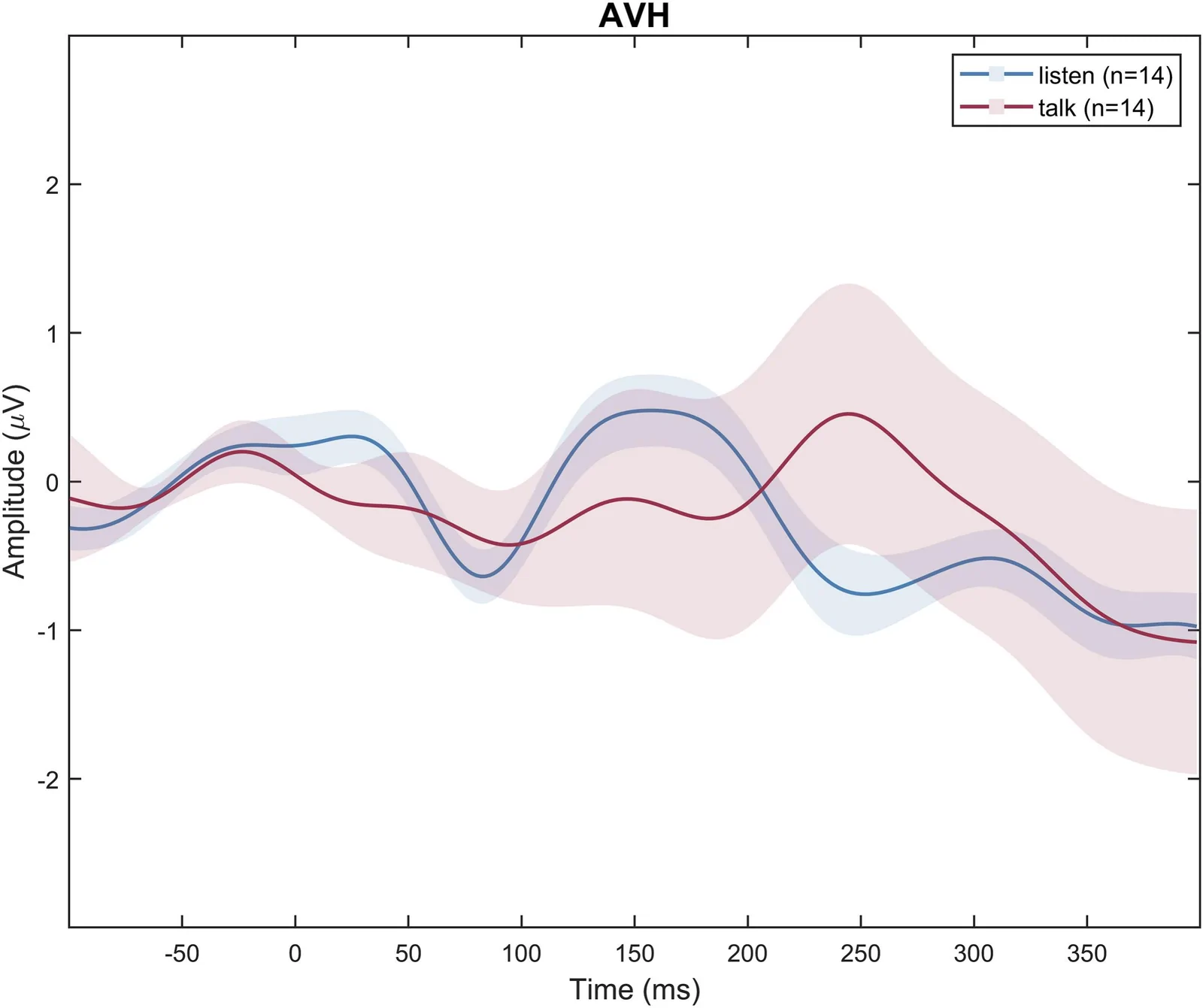

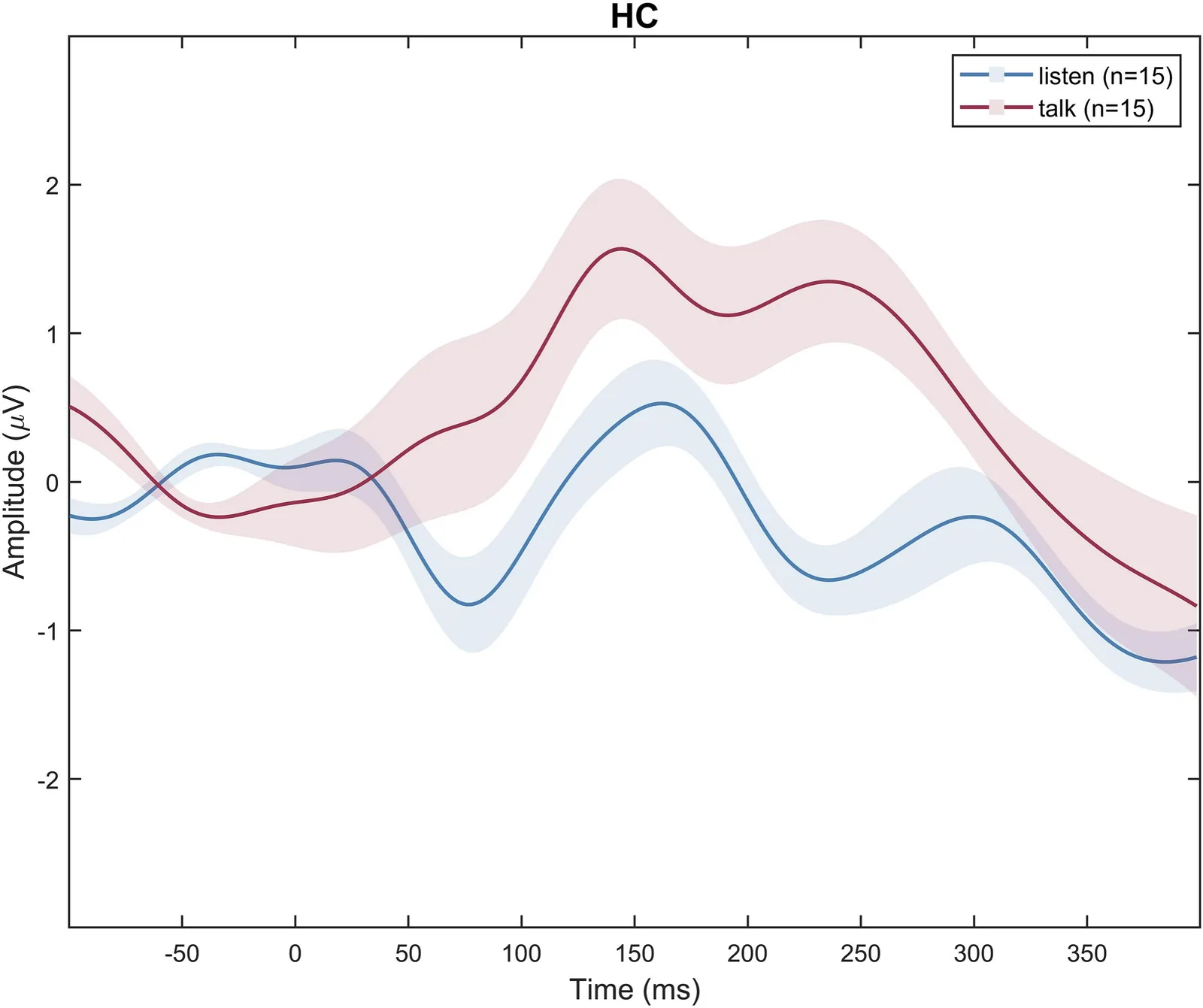

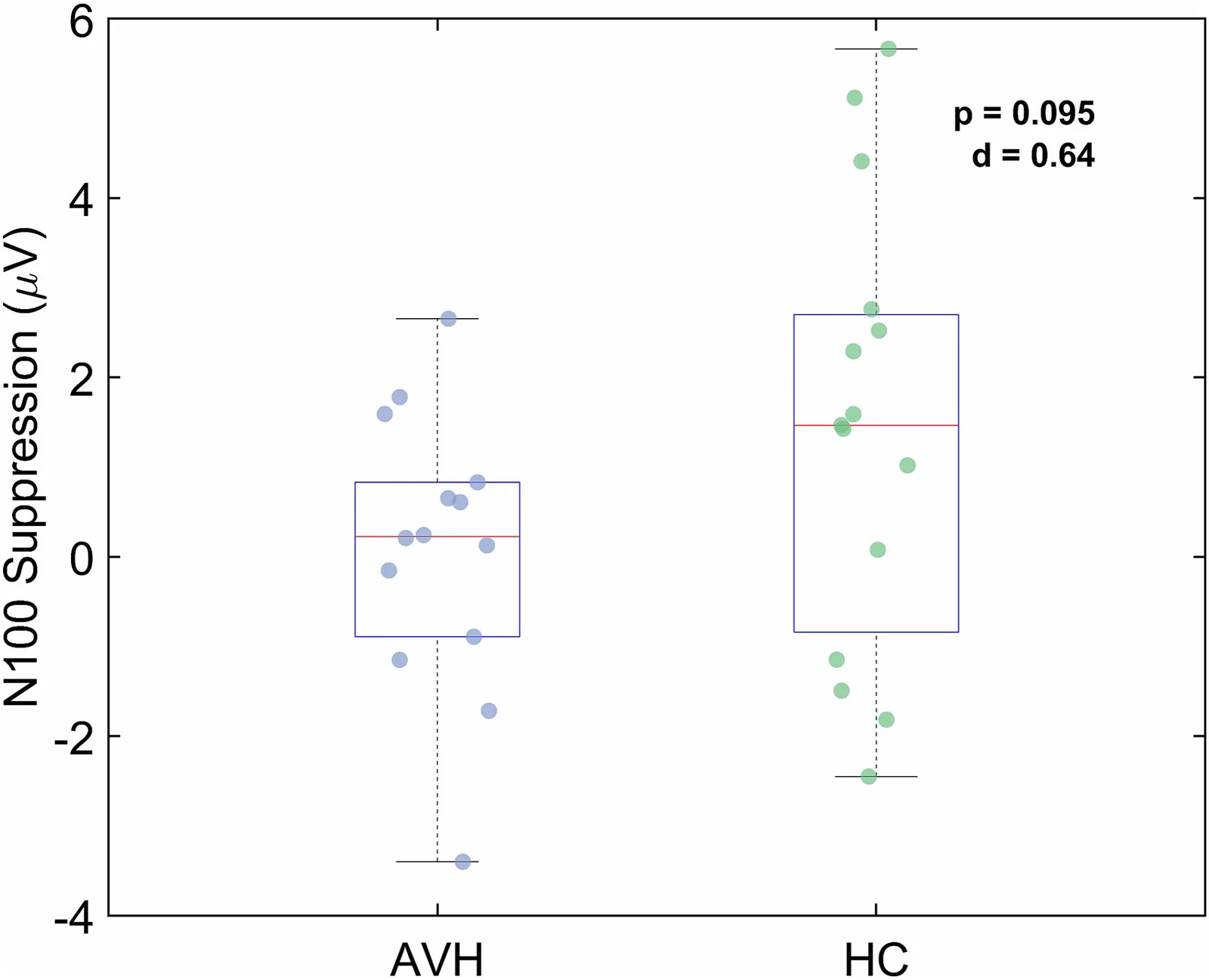
***Grand average ERPs and N100 suppression in AVH patients and healthy controls*** (**a**) Grand average event-related potential at fronto-central ROI (Fz-Cz) for AVH patients (*n* = 14). Shaded area represents standardized measurement error, (**b**) Grand average event-related potential at fronto-central ROI (Fz-Cz) for healthy controls (*n* = 15). Shaded area represents standardized measurement error, (**c**) Individual N100 suppression scores for AVH patients and healthy controls. Boxplots show median, interquartile range, and individual data points. *p* = 0.095, Cohen's d = 0.64.

### 3.3 Correlates with clinical symptoms and N100 suppression

Pearson's correlation test showed N100 suppression correlated significantly with hallucination severity in AVH patients (*r* = −0.54, *p* = 0.047), indicating that reduced suppression is associated with more severe auditory hallucinations (Fig. 2).

**Fig. 2:**
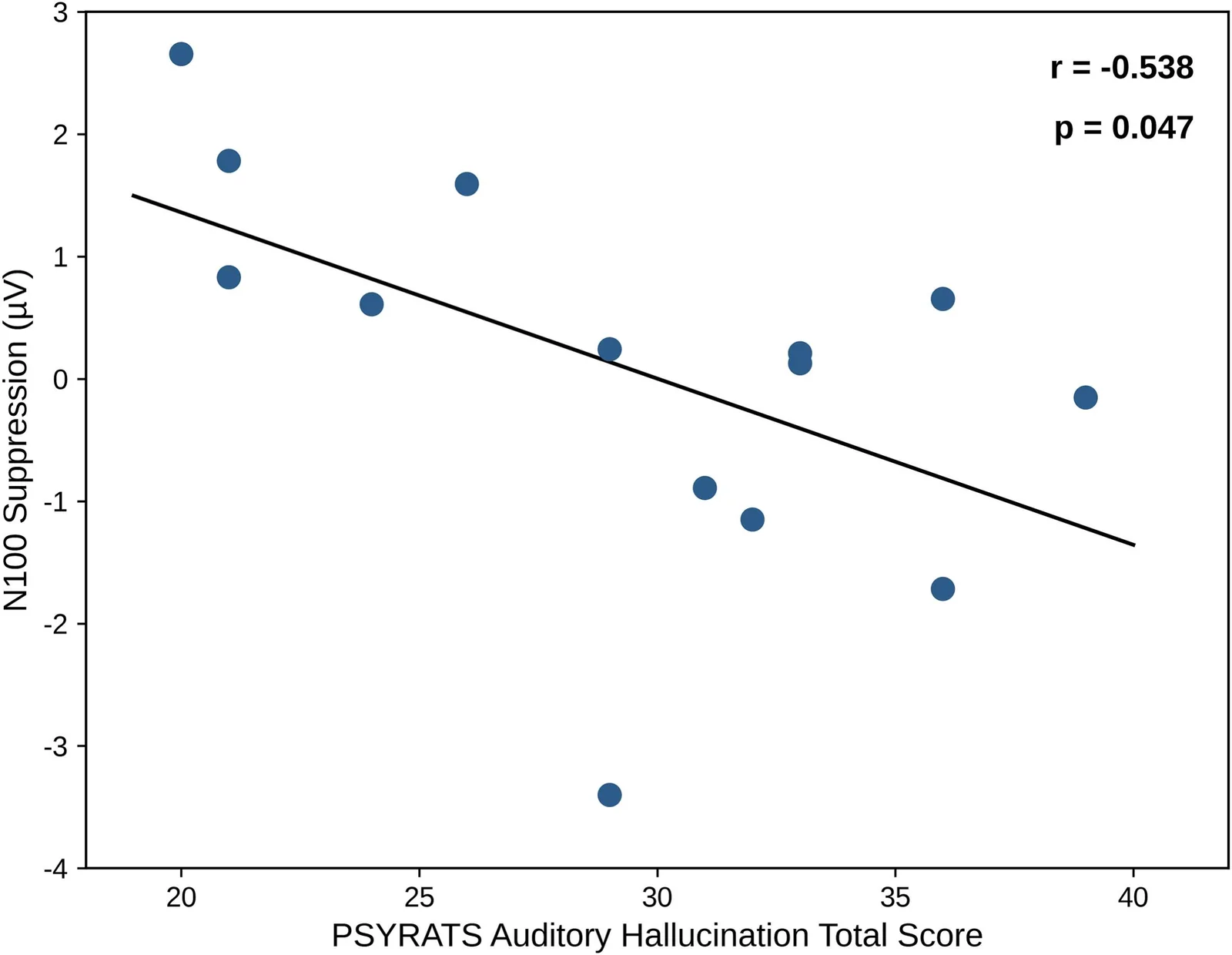
***Relationship between N100 suppression and hallucination severity*** Scatterplot showing the relationship between N100 suppression and PSYRATS-AHS total scores in AVH patients. Line represents linear regression fit.

## 4 Discussion

The present study investigated the corollary discharge mechanism in patients with persistent auditory verbal hallucinations using N100 suppression as a neurophysiological marker. A reduced N100 suppression in AVH patients compared to healthy controls was observed but not reaching statistical significance (*p* = 0.095, Cohen's d = 0.64). However, a significant negative correlation was found between N100 suppression and hallucination severity as measured by the PSYRATS-AHS (*r* = −0.54, *p* = 0.047), indicating that reduced suppression was associated with more severe auditory hallucinations.

The negative correlation between N100 suppression and PSYRATS-AHS scores represents a key, though preliminary, finding of this study. However, given the modest sample size (*n* = 14), this correlation should be interpreted with caution, as the estimate carries considerable uncertainty and could differ in a larger, independent sample. This supports the expected relationship that corollary discharge dysfunction is associated with the severity of AVH. Reduced N100 suppression reflects a failure to properly attenuate self-generated auditory signals, which may contribute to the misattribution of internal speech as externally generated voices. The strength of this correlation (*r* = −0.54) suggests a meaningful relationship between the neurophysiological mechanism and the clinical symptom.

Notably, this finding contrasts with prior research that has failed to find significant correlations between N100 suppression and hallucination severity. Multiple studies by Ford and colleagues found no significant correlations between N1 suppression and auditory hallucinations when using broad symptom rating scales including the BPRS and SAPS (Ford et al., 2007a; Ford & Mathalon, 2007). The discrepancy between these null findings and the current significant correlation may be explained by differences in symptom assessment. The PSYRATS-AHS captures multiple specific dimensions of auditory hallucinations, including frequency, loudness, and spatial. In contrast, broad rating scales like the BPRS and SAPS assess general psychotic symptomatology rather than detailed phenomenological characteristics of auditory hallucinations specifically. These findings suggest that AVH-specific rating scales such as the PSYRATS-AHS may be more sensitive to detecting relationships with neurophysiological markers of corollary discharge dysfunction. This highlights the importance of using symptom-specific measures in psychophysiological research, as they may reveal relationships that remain obscured when using broader assessment tools.

Regarding the between-group comparison, a trend toward reduced N100 suppression was observed in AVH patients compared to healthy controls (*p* = 0.095), with a moderate effect size (Cohen's d = 0.64). While this finding did not reach statistical significance, the effect size suggests a difference between groups that is consistent with previous literature demonstrating reduced N100 suppression in patients with schizophrenia (Ford et al., 2004; Ford et al., 2007a). The mechanisms underlying this finding likely relate to the effectiveness of the corollary discharge function. Greater N100 suppression reflects a more effective corollary discharge mechanism, in which the efference copy sent from motor to auditory cortical regions during self-generated speech successfully attenuates the expected sensory consequences of one's own vocalization (Ford and Mathalon, 2005). Reduced N100 suppression, as observed in the AVH group, is therefore thought to reflect a failure of this mechanism, whereby self-generated auditory signals are not adequately distinguished from externally generated ones. This is consistent with the established relationship between N100 suppression and AVH, supporting corollary discharge dysfunction as a potential underlying mechanism of auditory hallucinations (Parlikar et al., 2019; van Lutterveld et al., 2011). The failure to achieve statistical significance is likely attributable to the limited sample size (*n* = 14 AVH, *n* = 15 HC), which resulted from substantial data loss due to technical issues and excessive artifacts.

Semi-dry electrodes offer practical advantages over wet electrodes, including faster setup time and reduced technical demands, which can be particularly beneficial when working with psychiatric patient populations. Although impedances were maintained below 50 kΩ, significantly higher than the typical <5 kΩ target for wet electrodes, the data quality proved adequate for detecting N100 components. The use of bootstrapped standardized measurement error (bSME), as recommended by Luck et al. (2021), provides a standardized approach to quantifying data quality in ERP research. The bSME analysis revealed signal-to-measurement error ratios of 3.1 for the listen condition and 2.2 for the talk condition, indicating adequate signal quality despite the higher impedances. As expected, data quality was better in the listen condition compared to the talk condition, likely due to artifacts introduced by vocalization. These findings demonstrate that semi-dry electrodes can be successfully used for N100 suppression measurements.

Several limitations should be acknowledged. The primary limitation is the small sample size resulting from challenges in recruiting patients who met the criteria for AVH. These patients are severely ill and may have difficulty enrolling in and adhering to a clinical trial, which limited the study basis from the outset. Further, the cohort was reduced from the initially recruited 20 AVH patients to 14 due to technical issues (*n* = 3) and excessive artifact occurrence (*n* = 3). Similarly, 5 of 20 healthy control participants were lost due to technical issues. This substantial data loss, combined with greater variability in the healthy control group (SD = 2.49 μV) compared to the AVH group (SD = 1.54 μV), limited statistical power for detecting between-group differences, potentially contributing to the trend-level rather than significant finding in the group comparison. The higher impedances associated with semi-dry electrodes, while acceptable, may have contributed to increased artifact susceptibility compared to setups that allow for lower impedances. Additionally, antipsychotic medication doses were not controlled for in the analyses. In the present sample, 11 of 14 AVH patients were prescribed antipsychotics, of whom 5 were treated with clozapine specifically. EEG recordings can be influenced by antipsychotic medication; most notably, clozapine has been associated with EEG slowing in spectral analyses (Jackson and Seneviratne, 2019). The relationship between antipsychotic medication and N100 suppression specifically remains uncertain in the literature. Prior studies using a comparable talk/listen paradigm found no significant relationship between antipsychotic dose and N100 suppression, suggesting that medication effects on this specific measure may be limited (Beño-Ruiz-de-la-Sierra et al., 2023). Nevertheless, we cannot rule out that antipsychotic medication influenced our findings, and this should be considered when interpreting the results. Furthermore, it should also be noted that the presence of hallucinations other than AVH was not an inclusion or exclusion criterion, and screening for other hallucination modalities was not part of the study protocol. The focus of this study was specifically the AVH symptom, and AVH is by far the most common hallucination modality in schizophrenia spectrum disorders (Lim et al., 2016). However, this lack of information is still a limitation of the study, as the absence of systematic screening means we cannot report the rate or severity of other potential hallucination types in our sample.

Despite these limitations, the findings offer insights for both research and clinical practice. The significant correlation between N100 suppression and PSYRATS-AHS scores supports the use of detailed, symptom-specific assessment tools in psychophysiological research on auditory hallucinations. Future studies should prioritize the use of measures like the PSYRATS-AHS over broader symptom scales to maximize sensitivity for detecting relationships with neurophysiological markers. The feasibility of semi-dry electrodes demonstrated in this study suggests that corollary discharge research can be made more accessible to researchers with varying levels of ERP expertise and in diverse clinical settings.

This study provides preliminary evidence that auditory hallucination severity, as measured by the comprehensive PSYRATS-AHS, may be associated with impaired corollary discharge function reflected in reduced N100 suppression, warranting replication in larger samples. This finding underscores the importance of using symptom-specific assessment tools in psychophysiological research. While the between-group comparison showed only a trend toward significance, likely due to limited sample size, the moderate effect size is consistent with established literature. Additionally, the feasibility of semi-dry electrodes for N100 suppression measurements was demonstrated, potentially broadening the accessibility of this research methodology. Future studies with larger sample sizes are needed to replicate these findings and further investigate the relationship between corollary discharge dysfunction and the phenomenological characteristics of AVH.

## CRediT authorship contribution statement

**Ella Sonnelid:** Conceptualization, Formal analysis, Data curation, Visualization, Writing – original draft, Writing – review & editing. **Robert Boden:** Resources, Funding acquisition, Writing – review & editing, Supervision. **Caroline Wass:** Conceptualization, Writing – review & editing. **Jonas Persson:** Conceptualization, Data curation, Writing – review & editing, Supervision.

## Funding

The study was supported by grants from the Swedish 10.13039/501100000942Brain Foundation.

## Declaration of competing interest

The authors declare that they have no known competing financial interests or personal relationships that could have appeared to influence the work reported in this paper.

## Appendix A Supplementary data

Supplementary material Supplementary Figures S1 and S2. Figure S1: Study design and flow of participants, showing screening and baseline procedures, data processing steps, and participant attrition with reasons for exclusion. Figure S2: Photographs of the semi-dry electrode setup, showing (a) the EEG cap as placed on participants and (b) a close-up of the electrodes with sponges saturated in saline for coupling to the scalp.
