# Supplementary material for: Corollary discharge dysfunction in auditory verbal hallucinations: N100 suppression measured with semi-dry electrodes
Source: Clin Neurophysiol Pract. 2026 Oct 6;11:851–7. doi: 10.1016/j.cnp.2026.10.001 (PMC13675898; doi:10.1016/j.cnp.2026.10.001)
Supplement: Supplementary file 1 [file mmc1.pdf]

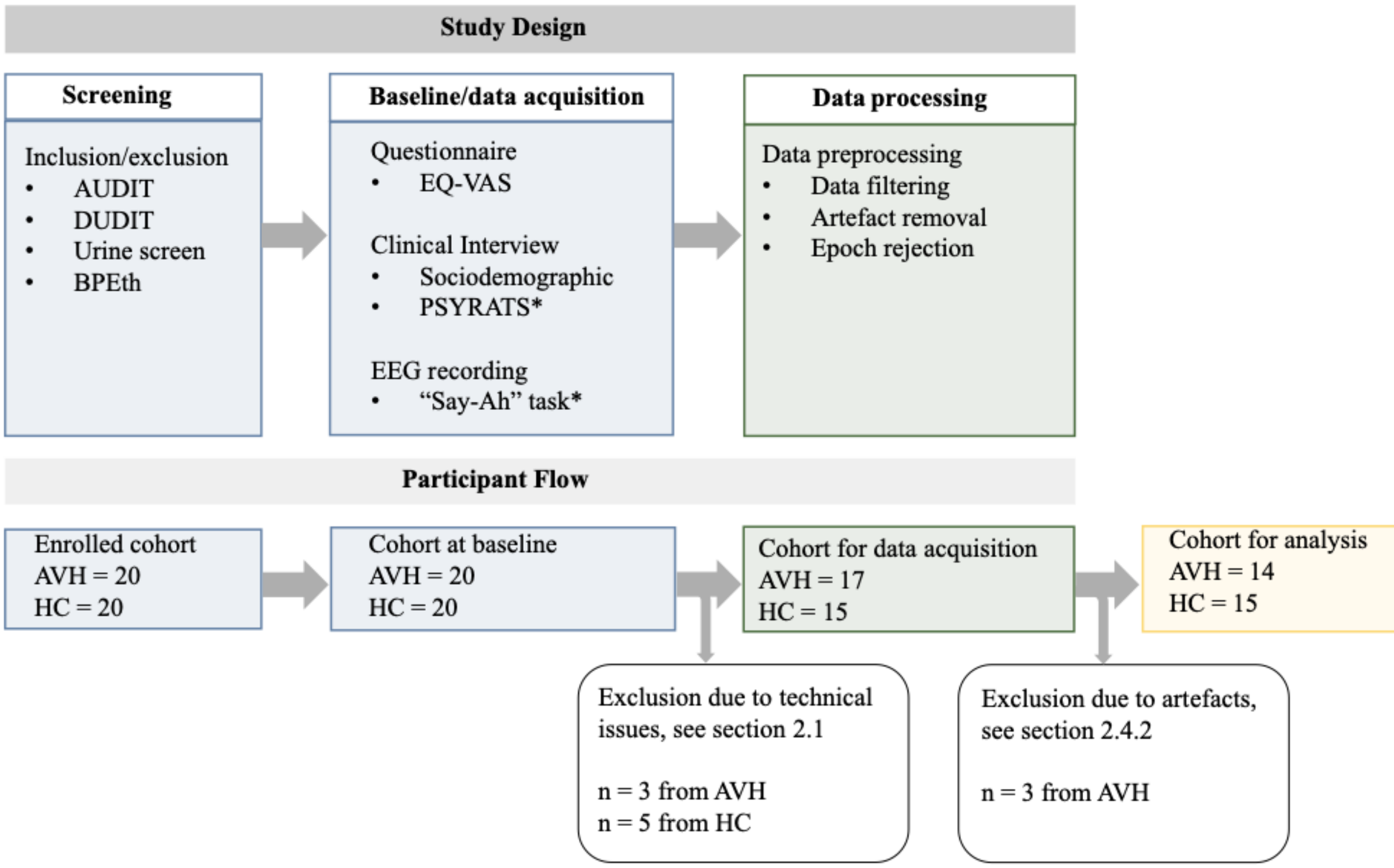

Figure S1. ***Study design and flow of participants***
AVH: patients recruited in the hallucination group. HC: healthy controls. * Primary outcome measure.

a) b)

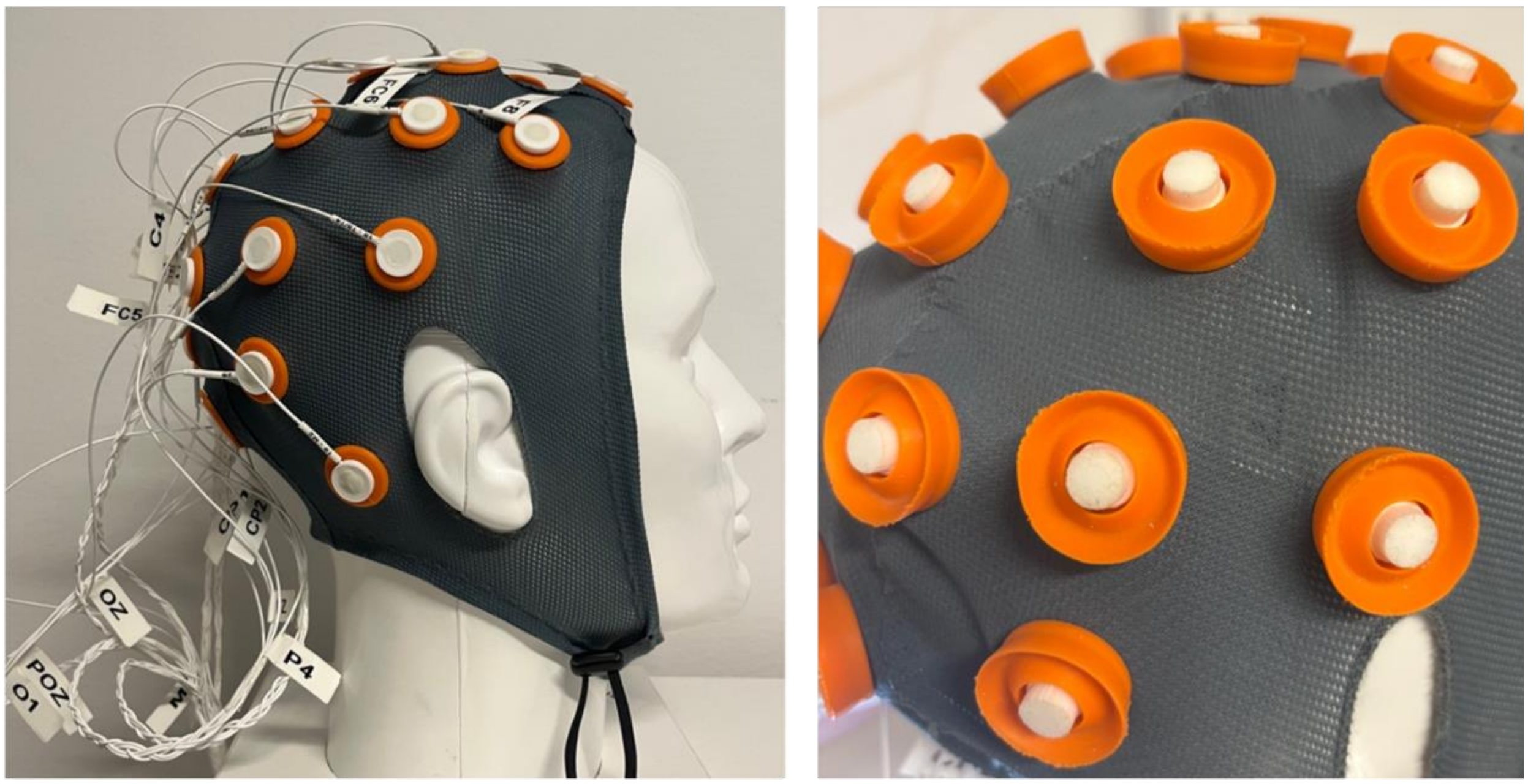

Figure S2. ***Images of semi-dry electrode setup, cap and electrodes***
(a) Image of cap places as it was on the participants, **(b)** Image of the inside of the electrodes with sponges in place, which were saturated in saline for coupling to the scalp.
